# Role of T cells in cancer immunotherapy: Opportunities and challenges

**DOI:** 10.1016/j.cpt.2022.12.002

**Published:** 2022-12-20

**Authors:** Hossain Ahmed, Aar Rafi Mahmud, Mohd. Faijanur - Rob - Siddiquee, Asif Shahriar, Partha Biswas, Md. Ebrahim Khalil Shimul, Shahlaa Zernaz Ahmed, Tanzila Ismail Ema, Nova Rahman, Md. Arif Khan, Md. Furkanur Rahaman Mizan, Talha Bin Emran

**Affiliations:** a Department of Biotechnology and Genetic Engineering, University of Development Alternative (UODA), 4/4B, Block A, Lalmatia, Dhaka, 1209, Bangladesh; b Department of Biochemistry and Molecular Biology, Mawlana Bhashani Science and Technology University, Tangail, 1902, Bangladesh; c Department of Biochemistry and Molecular Biology, Faculty of Biological Science, University of Dhaka, Dhaka, 1000, Bangladesh; d Department of Immunology and Microbiology, School of Medicine, University of Texas Rio Grande Valley, McAllen, TX, 78504, USA; e Department of Genetic Engineering and Biotechnology, Faculty of Biological Science and Technology, Jashore University of Science and Technology (JUST), Jashore, 7408, Bangladesh; f Department of Biochemistry and Microbiology, North South University, Dhaka, 1229, Bangladesh; g Department of Biochemistry and Molecular Biology, Jahangirnagar University, Savar, Dhaka, 1342, Bangladesh; h Department of Food Science and Technology, Chung-Ang University, Anseong, 17546, Republic of Korea; i Department of Pharmacy, BGC Trust University Bangladesh, Chittagong, 4381, Bangladesh; j Department of Pharmacy, Faculty of Allied Health Sciences, Daffodil International University, Dhaka, 1207, Bangladesh

**Keywords:** Immunotherapy, T cell, Cancer, Immune system, Metabolic pathways

## Abstract

Immunotherapies boosting the immune system's ability to target cancer cells are promising for the treatment of various tumor types, yet clinical responses differ among patients and cancers. Recently, there has been increasing interest in novel cancer immunotherapy practices aimed at triggering T cell-mediated anti-tumor responses. Antigen-directed cytotoxicity mediated by T lymphocytes has become a central focal point in the battle against cancer utilizing the immune system. The molecular and cellular mechanisms involved in the actions of T lymphocytes have directed new therapeutic approaches in cancer immunotherapy, including checkpoint blockade, adoptive and chimeric antigen receptor (CAR) T cell therapy, and cancer vaccinology. This review addresses all the strategies targeting tumor pathogenesis, including metabolic pathways, to evaluate the clinical significance of current and future immunotherapies for patients with cancer, which are further engaged in T cell activation, differentiation, and response against tumors.

## Highlights

• Immunotherapies boost the immune system's power to target cancer cells.
• Immunotherapy is a groundbreaking research area in cancer treatment.
• Cancer immunotherapy practices aim to trigger T cell-mediated anti-tumor responses.
• We discussed strategies to target metabolic pathways to improve immunotherapies.

## Graphical abstract

**Figure:**
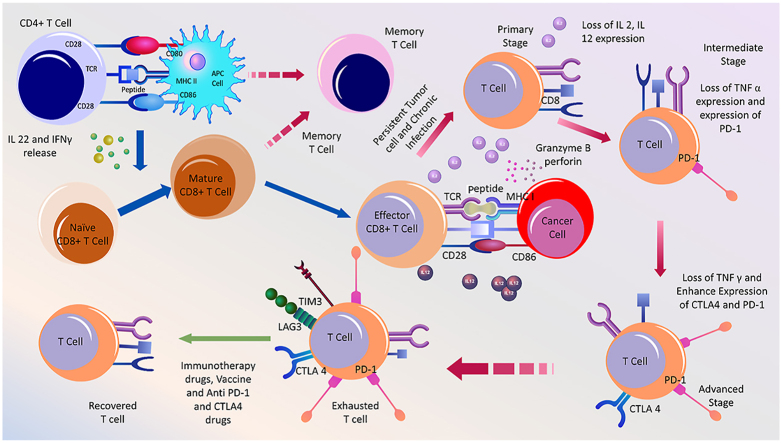

## Introduction

The global cancer-related death rate from 2007 to 2017 shows a sharp increase of 25.4%, and the number of cancer diagnoses and the worldwide rate of death were 18.1 and 9.6 million in 2018, respectively.^1^ Cancer development and endurance are caused by multiple factors, including somatic cell mutations and physiological alterations in cancer cells and the immune system.^2^ Cellular changes that lead to carcinogenesis, uncontrollable cell proliferation, angiogenesis, immune system dysfunction, and immunoediting can be caused by the presence or absence of functional mutations in oncogenes and tumor suppressor genes, respectively.^3^ Cancer is classified into two histopathological forms: metastatic and non-metastatic. Over the years, increasing knowledge of cancer immunology has led to the prevention and control of cancer cell immune evasion, as reported by successful clinical trials.^2^ Strategies include hormonal therapy, chemotherapy, radiation therapy, immunotherapy, and surgery. Evidence supports the use of combination rather than single treatments due to better outcomes, particularly for more advanced and progressive cancers.^4^ Cytotoxic lymphocytes, such as CD8+ and CD4+ cells, alongside their respective cytokine interferon-gamma (IFN-γ), play a significant role as tumor antagonist effectors. Fast and specific recognition of tumor-specific antigens by immune T cells is the foundation of cancer immunology and cancer immunotherapy.^5^ As T cells travel around the body, they scan for major histocompatibility complex (MHC)-class peptide complexes that induce T cell receptors (TCRs). Tumor-defined T cells are activated when they encounter tumor antigens presented by antigen-presenting cells (APCs) such as dendritic cells (DCs). Additionally, T lymphocytes can directly identify antigens displayed on the surface of tumor cells.^6^ Several studies have shown that CD4+ cells are vital for priming MHC I-restricted CD8+ cytotoxic lymphocytes to attain full activation and effector function. Studies using mouse models have demonstrated that diminishing CD4+ cells results in tumor rejection failure by the cellular defense system.^7^ Furthermore, CD4+ cells support antigen specificity for effector cells, with no antigen-specific recognition capacity. Therefore, MHC I-restricted CD8+ cells, which specifically lyse tumor cells *in vitro*, are candidates for clinical tumor vaccine trials.^5^^,^^8^

Adoptive cell transfer (ACT) immunotherapy has been reported as an effective strategy against cancer. Usually, tumors persist despite the enrichment of tumor-specific T cells in tumor-infected areas.^4^ In this regard, scientists hypothesize that T cells within the tumor microenvironment (TME) experience chronic activation and are submerged with immunosuppressive molecules, which affects their protective function.^6^ Clonal expansion occurs when these tumor-infiltrating lymphocytes (TILs) are extracted from the TME and cultured and expanded *ex vivo*. During this process, extracted tumor cells and TILs are eradicated by activated natural killer (NK) cells or newly synthesized T cell populations. Following further expansion, these cells are transferred back to the host, where tumor cell death and complete eradication occur.^6^

Over the years, scientists and physicians have studied the duality of the immune system to protect the host while also contributing to tumor development that can be achieved via immunosurveillance and immunoediting. Immunosurveillance occurs when the defense system eliminates microscopic but potential tumors before they emerge into established tumors. However, the immune system can alter the mRNA expression of an efflorescing tumor, where the immune-responsive antigenic portions of the molecule are edited or completely removed, meaning that tumors can escape recognition by CD4+ cells.^8^ Furthermore, tumor-associated macrophages (TAM) or myeloid-derived suppressive cells (MDSC), along with their secreted cytokines interleukin (IL)-6, tumor necrosis factor (TNF), and IL-1β, contribute to the promotion of cancer development and progression to advanced stages.^3^

## Cancer immunotherapy

The mechanism of cancer immunotherapy involves three steps. First, APCs, such as DCs, attack antigens in cancer cells and break them into antigenic peptides. These peptides are found in human leukocyte antigens (HLA).^9^ In the second step, stimulating T cells are essential for the attachment of surface molecules, B7 and CD28, found in APCs and T cells, respectively.^10^ Both signals are required for the maximum activity of T cell signaling. However, antigenic peptide formation is impossible without molecular stimulation by the first signal. Therefore, the absence of antigenic peptides leads to incomplete activation of T cells, inducing T cell tolerance.^10^ Inhibitory molecules are also present in this mechanism: cytotoxic T-lymphocyte–associated antigen 4 (CTLA-4) and programmed death 1 (PD-1) induce signals to prevent T cell activation. Finally, stimulated cancer-specific T cells arrive at the tumor site and recognize the tumor antigens expressed by cancer cells, eventually killing the cancer cells. While antigenic peptides are responsible for T cell stimulation, T cell activation is associated with co-stimulation and co-suppression.^11^ Recent clinical trials have shown that blocking PD-1 co-inhibition with anti-PD-1 or anti-programmed death ligand-1 (anti-PD-L1) therapy can stimulate T cell-mediated anticancer actions without severe consequences.

## Role of T cells in cancer

The human body is continuously scanned by DCs. When they encounter secreted tumor-specific antigens, the antigens are phagocytized and displayed on the surface of MHC class II molecules. Later, when DCs enter the secondary lymph nodes, MHC class II peptides are identified by T cells and naïve T cells, including cytotoxic T lymphocytes (CTLs).^12^ T cells undergo maturation and activation, reach effector status, and are released to scan for and eliminate their target cancer cells.^13^ Naïve CD4+ T cells can differentiate into several T helper (Th) cells, including Th1, Th2, Th9, Th17, and T regulatory cells (Tregs).^14^ Based on the specific cytokines released by the CD4+ cells, they are categorized as Th1 or Th2 cells. It has been theorized that an imbalance between Th1 and Th2 cells leads to a dysfunctional immune system, resulting in autoimmune disease development.^15^ In contrast, Th17 cells are novel CD4+ T helper cells that secrete IL-17 and protect the host from pathogens. However, excess Th17 cells can induce autoimmune diseases and inflammation. Tregs maintain immune system homeostasis and prevent cytokine storms.

Interestingly, Tregs and Th17 cells share some standard functions.^16^ Thirty years of research have shown that CD4+ T cells are significant participants in human tumor rejection. They can support CD8+-mediated tumor elimination via priming cytotoxic CD8+ T cells. Additionally, a subset of CD4+ T cells can directly participate in tumor cell elimination.^17^ CD8+ T cells are differentiated cytotoxic lymphocytes that act as effector molecules in the inactivation of CD4+ T cells. However, they do not exhibit an effector-memory function, may have reduced effectiveness in fighting off tumor cells, and become more prone to fatigue in tumor rejection,^13^ indicating that CD4+ T cells induce the activation and maturation of CD8+ T cells and maintain their effectiveness and ability to transform into effector memory CD8+ T cells.^18^

When CD4+ T cells encounter MHC class II molecules, IFN-γ is released, stimulating host cells to eradicate tumor cells. IFN-γ induces the secretion of two CXC chemokines, Mig and interferon-gamma inducible protein 10 kDa (IP-10). These chemokines exhibit anti-angiogenic actions by destroying the tumor vasculature, leading to growth inhibition and tumor necrosis. In addition, IFN-γ activates macrophages to release TNF and nitric oxide.^19^ However, IFN-γ is insufficient in the absence of antigen-stimulated CD4+ T cells in the TME because the synergistic action of CD4+ T cells and IFN-γ activates TNF, IP-10, Mig, and other tumoricidal cells.^17^

### Current progress in T cell-based cancer immunotherapy

Immunotherapy, in which cancer cells are eradicated using the patient's immune system, forms a groundbreaking research area in cancer treatment. These therapies were shown to eliminate tumors successfully in clinical trials.^20^ Cancer-based immunotherapy requires stimulation and expansion of cancer-specific T cells, which function by recognizing the antigens in cancer cells and thereby eradicating cancer.^21^

### Adoptive T cell therapy

In ACT, T cells are directly used in targeting cancer. The ACT strategy can be completed in three stages. TILs are obtained from patient tumor samples, followed by *in vitro* expansion and modification.^22^ Subsequently, the cells are transfused to the patient.

TILs may contain numerous tumor-reactive T cells, ultimately forming polyclonal therapeutic cells. Several studies have shown that TILs play a vital role in melanoma.^23^ The primary concern of ACT therapy is the probability of delayed side effects. Graft-versus-host disease (GVHD) was initially observed during bone marrow transplantation,^24^ and the concern for the potential side effects remains.

### Chimeric antigen receptor T cell therapy

Chimeric antigen receptor (CAR) T cell therapy comprises CAR T cells as a vital component. This therapy is based on providing machinery to T cells enabling them to distinguish tumor antigens that do not require HLA and activate them to recognize more extensive target antigens at a higher efficacy than the natural TCR.^25^ CAR consists of a segment that recognizes the tumor-associated antigen (TAA) and two activation domains responsible for lymphocyte stimulation.^26^ T cell stimulation can be achieved through CAR mediation, which is only feasible when a tyrosine activation motif is present.^27^ However, there are many obstacles associated with CAR T cell therapy. The cells should accumulate at tumor sites to properly attach the target proteins to tumor surfaces. This is essential for the systematic and structured functioning of T cell immunotherapy, but limitations are posed by an immunosuppressive environment.^28^ The absence of chemokine receptors in T cells can make it challenging to conduct cell trafficking and infiltrate cells into tumor sites, hindering the efficacy of CAR T cells.^16^ Therefore, T cells must be modified so that chemokine receptors match the corresponding chemokines present in tumor cells.^29^

## Role of CD4+ T cells in cancer immunotherapy

Previous research has focused on CD8+ T cells to eradicate cancer because of their tumor cell recognition via MHC class-1 receptor complexes and the potential to lyse tumor cells directly upon recognition.^30^ However, many studies have revealed that CD8+ T cells use CD4+ T cells for their effector activity and sustain functional potential^31^ with the involvement of APCs.^32^ CD8+ T cell effector activity, proliferation, and recruitment to the tumor site are largely augmented by tumor-specific CD4+ T cells via IL-2 signaling.^33^ Additionally, CD4+ T cell responses promote the secondary expansion of CD8+ T cells and memory T cell creation.^34^ Furthermore, CD4+ cells directly kill tumor cells via the secretion of effector molecules, such as cytokines (IFN-γ and TNF-α), upon DC activation.^35^

As aforementioned, naïve CD4+ T cells can differentiate into many subsets (Th1, Th2, Th9, Th22, Treg, Th17, and Tfh) according to their interaction with DCs and exposure to cytokines.^36^ Differentiation into these lineages occurs via complicated but specific cytokine signaling and transcription factors regulated by epigenetic modifications.^37^ The effects of Th1, Th2, Th17, and Tregs on anti-tumor immunity have been well characterized. IL-12 and IFN-γ trigger the development of Th1n, activation of a downstream signaling cascade, and the coordination of several transcription factors such as T-bet, signal transducer and activator of transcription-1 (STAT1), STAT4, Runx 3, Eomesodermin (Eomes), and Hlx, which act as eliminators of intracellular pathogens.^37^ Th1 cells tend to produce IL-2, which enhances CD8+ memory T cell development after antigen priming.^37^ IL-4 and IL-2 induce Th2 priming from naïve T cells, whereas STAT6, which is induced by IL-4, augments expression.^37^ GATA binding protein 3 (GATA3) promotes IL-4 production, while IL-5 and IL-13 are associated with the elimination of helminth parasites. IL-17A, IL-17F, and IL-22 are signature cytokines that characterize Th17 cells.^38^ In addition, IL-1β, IL-23, and transforming growth factor beta (TGF-β), which induce the expression of retinoic acid receptor-related orphan nuclear receptor gamma (RORγt) in humans, are crucial for Th17 cell differentiation.^36^ The role of Th17 in promoting or inhibiting malignancy is dependent on the tumor phenotype.^39^

### CD4+ T cells in anti-tumor immunity

A study of patients with melanoma showed that CD4+ T cells frequently recognize mutant neoantigens.^40^ Moreover, the immunogenic mutanome of non-synonymous cancer mutations was predominantly recognized through CD4+ T cells, but not CD8+ T cells, in three mouse tumor models.^41^ Additionally, the administration of CD4+ cytotoxic T cells can lead to an anti-tumor response. Cytotoxic CD4+ subsets are clonally expanded in bladder tumors, possibly due to tumor antigen recognition. These CD4+ subsets have autologous tumor cell killing abilities (as they secrete TNF-α and IFN-γ) *ex vivo.*

Single-cell analysis revealed a gene signature of proliferating and non-proliferating CD4+ cytotoxic T cells, supporting the use of anti-PD-L1 therapy in inflamed metastatic bladder cancer in an independent set of 62 patients.^42^ Analysis of the immune response of Th1-type CD4+ T cells against human papillomavirus type 16 (HPV16) E2, E6, and E7 in disease-free women exposed to robust E2-and E6-specific proliferation-associated reactions associated with the secretion of IFN-γ and IL5.^43^ However, the study did not show any direct involvement in tumor control.

RNA sequencing analysis of single T cells together with TCR tracking from colorectal cancer biopsies has demonstrated that the CXCL13+ BHLHE40+ Th1 subset of CD4+ T cells shows preferential enrichment with microsatellite-unstable tumors. This offers a probable explanation for the favorable response of these tumors to immune checkpoint blockade (ICB).^44^ Furthermore, highly immune CD4+ T cells that recognize COA- 1, telomerase reverse transcriptase (TERT), and mesothelin-derived peptides have been shown in patients with chemotherapy-naïve metastatic colorectal cancer (mCRC) and CD4+ T cells have been shown to sustain their anti-tumor functions for three months during oxaliplatin treatment.^45^

Experiments were performed using mice bearing sarcoma cells. mLAMA4 tumors have indicated that CD4+ T cells amplify CD8+ T cell priming and their maturation into CTLs.^46^ IL-21 is secreted by CD4+ T cells, directing the differentiation of cytolytic CX3CR1+CD8+ T cells (a subset of CD8+ T cells) to protect against persistent viral infection and exert anti-tumor activity.^47^ Additionally, a study on B16 melanoma revealed that IL-21+ CD4 T cells induce a greater than 2-fold increase in CX3CR1+CD8 TILs.^47^ These findings may be useful in cancer immunotherapy.

The clinical prognosis of CD4+ T cells from the blood of patients with cancer, together with the assessment of tumor cell-directed CD4+ T cells, can reveal the relevance of CD4+ T cell responses in cancer immunotherapy. A higher anti-TERT Th1 response and low expression of PD-1+ T cell immunoglobulin (Ig) mucin-3 (TIM-3)+ CD4+ T exhausted cells are associated with better patient prognosis in non-small cell lung cancer (NSCLC). Furthermore, the systemic anti-TERT Th1 response drives robust possessive anti-tumor activity in NSCLC.^48^ Peripheral blood from a patient with NSCLC revealed that the responder carried a high proportion of effector CD62L-low CD4+ T cells prior to the PD-1 blockade. In contrast, non-responders had a higher proportion of CD25+ FOXP3+ CD4+ T cells. Interestingly, mass cytometry analysis revealed that the CD62L-low CD4+ T cell subset expressed T-bet+, CD27, FOXP3-, and CXCR3+.^49^ CD4+ T cells that recognize Melan-A have been linked to shortened survival in a cohort of preselected patients with Melan-A and/or NY-ESO-1 reactivity. Additionally, IL-4 and IL-17 expression in CD4+ T cells following Melan-A stimulation is negatively associated with patient survival.^50^

### CD4+ T cell-mediated cancer immunotherapy

**Figure 1:**
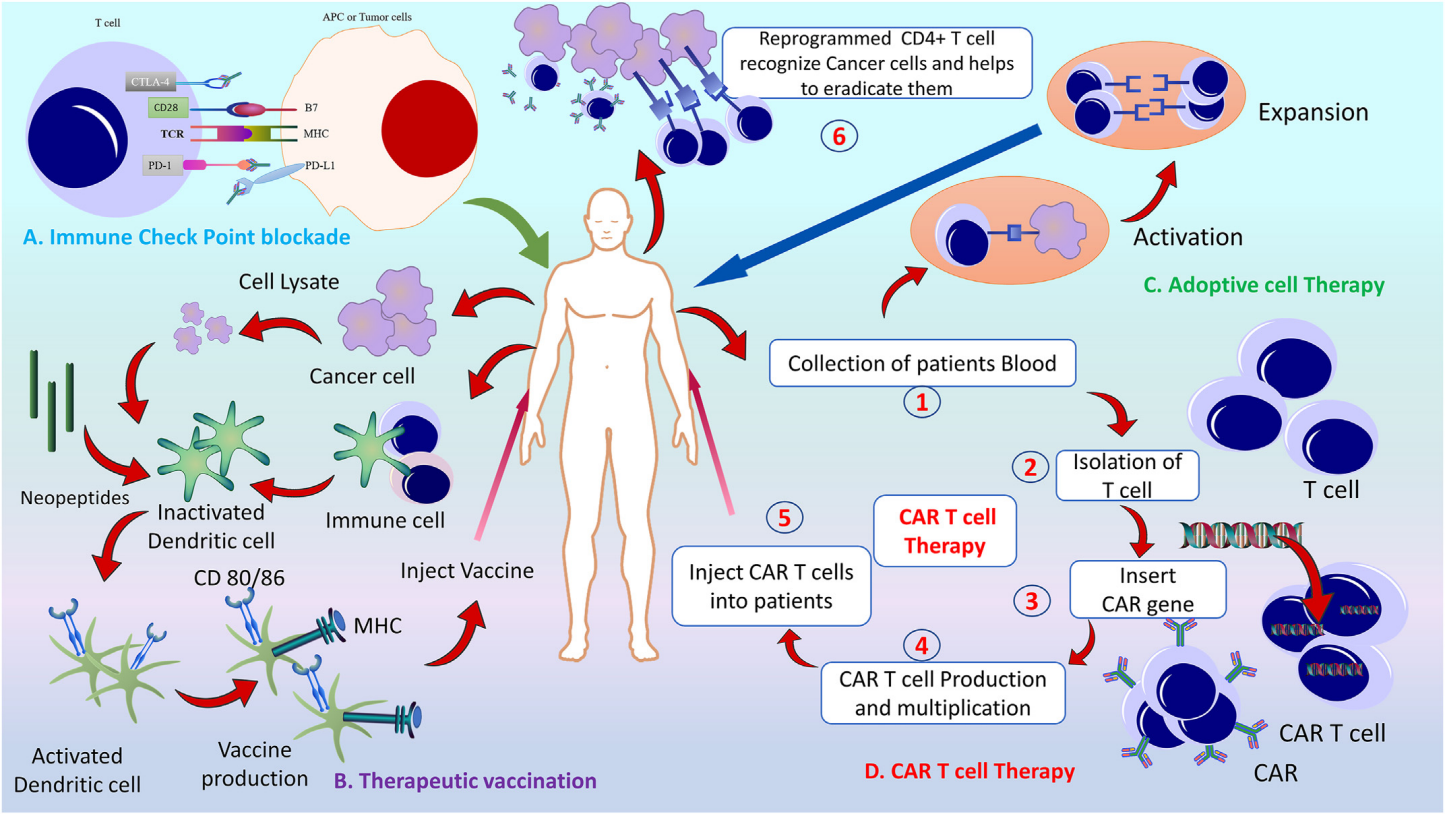
Action of a potential CD4+ T cell-based immunotherapy strategy against cancer. CD4+ T cells have a vital role in cancer and cancer immunotherapy. Immune checkpoint inhibitors are a potential strategy for cancer cells to bypass immune cell attacks. CD4+ T cells can be primed through therapeutic vaccination via generalized or personalized vaccines. These are designed according to patients' white blood and particular cancer cells. Blood is collected and reprogrammed through adoptive cell therapy and individual CAR T cell therapy. This cell therapy can mediate more specific attacks against cancer cell lines in CD4+ T cells. CAR genes are introduced into CD4+ T cell lines and amplified. Reprogrammed CAR T cells are administered into the patient's blood. In adoptive cell therapy, engineered CD4+ T cell lines are activated via cancer or antigen-presenting cells to increase the immune response. CAR: Chimeric antigen receptor.

Multiple approaches are used in cancer immunotherapy, including ICB, therapeutic vaccines, ACT approaches for TILs, and genetically engineered CAR T cells [Figure 1]. However, natural and immunotherapy-induced anti-tumor responses depend on tumor antigen-specific CD8+ and CD4+ T cell activity. Furthermore, CD4+ T cells must be activated within the TME.^46^ Therefore, harnessing the full potential of CD4+ and CD8+ T cells is an increasing necessity in cancer immunotherapy.

#### Immune checkpoint blockade

Tumor cells express ligands for T cell inhibitory receptors to escape immune surveillance and responses. Therefore, checkpoint inhibitor (CPI) antibodies are promising candidates for cancer immunotherapies.

Many experiments have assessed the potential role of CPI antibodies in the T cell immune response. Comprehensive profiling of the effect of checkpoint blockade against tumor immune infiltrates in mouse tumor models and human melanoma has demonstrated that anti-PD-1 and anti-CTLA-4 induce exhausted CD8+ T cell subsets. Conversely, CTLA-4 blockade promotes the expansion of effector CD4+ T cells with an inducible co-stimulator (ICOS+) Th-1 phenotype.^51^ Similarly, the blood and tumor tissues of patients with bladder cancer treated with anti-CTLA-4 antibodies showed a higher expression of ICOS CD4+ T cells. In addition, these T cells secrete IFN-γ, which recognizes NY-ESO-1.^52^ NY-ESO-1 antigen-specific CD4+ T cells directly lysed autologous melanoma cells, and their responses were enhanced after ipilimumab treatment. Upon NY-ESO-1 recognition, these CD4+ T cells expressed the master transcription factor Eomes.^53^

After ipilimumab (anti-CTLA-4) treatment, patients with melanoma had an increased absolute lymphocyte count (ALC) and delayed enhancement of CD4+ and CD8+ T cells, which was associated with a positive outcome.^54^ Additionally, biopsies from patients after treatment with pembrolizumab (an antibody against PD-1) and an investigation of single-cell infiltrates demonstrated an increase in CD4+ effector T cells in non-responding tumors due to therapy. In contrast, the frequency of CD4+ effector memory T cells (Tem) reduced during treatment.^55^

#### Therapeutic vaccination targeting of CD4+ T cells

A synthetic long-peptide vaccine against HPV-16 oncoproteins, E6 and E7, has shown a clinical response in patients with HPV-16–positive, third-grade vulvar intraepithelial neoplasia. This vaccine induced robust IFN-γ-associated responses in CD8+ and CD4+ T cells. In contrast, HPV-specific CD4+ T cells showed higher expression of IFN-γ in patients with a complete response than in patients who did not respond to treatment.^56^ The frequency of HPV16-specific CD4+ CD25+ Foxp3+ T cells was higher in the patient group with more extensive lesions; however, after vaccination, there was a decrease in the HPV16-specific IFN-γ/IL-10 ratio.^57^ CD4+ and CD8+ T cells can be recruited into the TME by sipuleucel-T (an approved autologous cellular vaccine against metastatic castration-resistant prostate cancer) treatment.^58^ This treatment is associated with the enhanced expression of Th1-associated genes but not Th2, Th17, or Treg cells. In addition, a reduction in serum prostate-specific antigen (PSA) is linked to Th1 response induction, whereas an increase has been linked to immune checkpoint protein induction following treatment.^59^

A phase 1/2 trial of a long synthetic peptide vaccine targeting prostate cancer induced a robust CD4+ response. This immunization was safe, tolerable, and long-lasting.^60^ The combination of NEO-PV-01 (a personalized neoantigen-based vaccine) and PD-1 blockade was shown to be safe in a phase 1b trial against multiple cancers, including advanced melanoma, NSCLC, and bladder cancer. The vaccine induced *de novo* neoantigen-specific responses in CD4+ and CD8+ T cells in all patients, trafficking these cells to the tumors to kill them.^61^

#### Adoptive transfer of genetically modified CD4+ helper T cells

ACT using TILs is a prominent approach in cancer immunotherapy. Many studies in animals and humans have been conducted with cytotoxic CD8+ effector TILs, and their roles have been well-studied. Recent studies have attempted to elucidate the potential impact of CD4+ TILs. The adoptive transfer approach of combined CD4+ and CD8+ T effector cells in mouse metastasis models demonstrated the critical role of CD4+ T cells in intensifying CD8+ T cell function. CD8+ effector cells have poor infiltration rates in tumors, whereas CD4+ T effector cells show high infiltration rates while simultaneously stimulating tumor antigen-specific CD8+ T cells.^62^ Flow cytometric analysis of TILs confirmed that at least 20% of metastatic melanomas accommodate CD4+ anti-tumor effector cells with specific tumor recognition.^63^ Patients with acral melanoma with non-synonymous mutations have shown a sustained response to TIL therapy via a CD4+ helper T cell response to oncogenic mutated BRAFV600E.^64^ Similarly, TILs from cholangiocarcinoma patients containing Th1 CD4+ subsets recognize a cancer-expressed mutated erbb2-interacting protein (ERBB2IP), as shown by the whole-exome-sequencing-based approach. These mutation-specific polyfunctional Th1 type CD4+ cells gained tumor control when the ACT approach with 25% TILs was used. Interestingly, consequent to disease progression, the administration of >95% pure population of mutation-reactive Th1 type CD4+ cells further induced tumor regression in a patient.^23^ Additionally, infusion of NY-ESO-1-specific autologous CD4+ T cell clones into a patient with refractory metastatic melanoma promoted enduring clinical relief and showed a response to other melanoma antigens, excluding NY-ESO-1.^65^

#### CD4+ chimeric antigen receptor T cell-mediated anti-tumor functionality

A higher proportion of CD8+ CD45RO- CD27+ memory T cells and a higher CD4+/CD8+ ratio in leukapheresis products created to generate T cells against multiple myeloma are linked to improved expansion *in vivo* and a positive clinical response in patients.^66^ However, the role of CD4+ T cells in the anti-tumor response has not been elucidated. Additionally, CD4+ CAR T cells showed persistent tumor challenge and effective function, whereas CD8+ CAR T cells were exhausted immediately after expressing their effective function following stimulation with IL13Rα2+ glioblastoma (GBM) cells. Moreover, CD4+ CAR T cell maintenance is positively associated with the recurrent killing capability of CAR T cells in GBM patients.^67^ As CD8+ cells are prone to exhaustion, CD8+ CAR T cells, particularly those with a high load of the target antigen, may be surpassed by CD4+ CAR T cells. In addition, preclinical analysis of murine tumors has demonstrated that the administration of CD4-LV exerts a faster and higher caliber killing of tumor cells than CD8-LV administration alone or in combination with CD4-LV.^68^ *In vivo*, CD4+ T cells co-expressing CAR T cells unique to B7H6 mouse tumors and overexpressing T-bet promoted anti-tumor responses and lengthened the survival of RMA-B7H6 lymphoma-bearing mice.^69^ Interestingly, CD19-targeted CAR T cell therapy in an immunocompetent, syngeneic mouse model of pre–B cell acute lymphoblastic leukemia showed a reduction in CD8+ CAR T cell efficacy, which was linked to T cell exhaustion and apoptosis. In contrast, CD4+ CAR T cells exhibit a cytotoxic function similar to that of CD8+ CAR T cells and retain *in vivo* efficacy even with TCR stimulation.^70^ A study based on single-cell transcriptional analysis and anti-CD19/4-1BB/CD28/CD3f CAR T cell cytokine signatures in antigen-specific stimulation revealed that CD4+ and CD8+ CAR T cells have equivalent cytotoxic functions. Additionally, their cytotoxic activity is linked to an elevated spectrum of Th1 and Th2 signature cytokines (such as IFN-γ, TNF-α, IL5, and IL13) via the expression of TBX21 and GATA3.^71^

*In vitro*, CD4+ CAR T cells demonstrated similar cytotoxicity to CD8+ CAR T cells against tumor cells, albeit at a lower level. However, higher levels of growth upon contact with tumor cells and increased IFN-γ and TNF-α levels were observed.^72^

## CD8+ cytotoxic T lymphocytes in cancer immunotherapy

**Figure 2:**
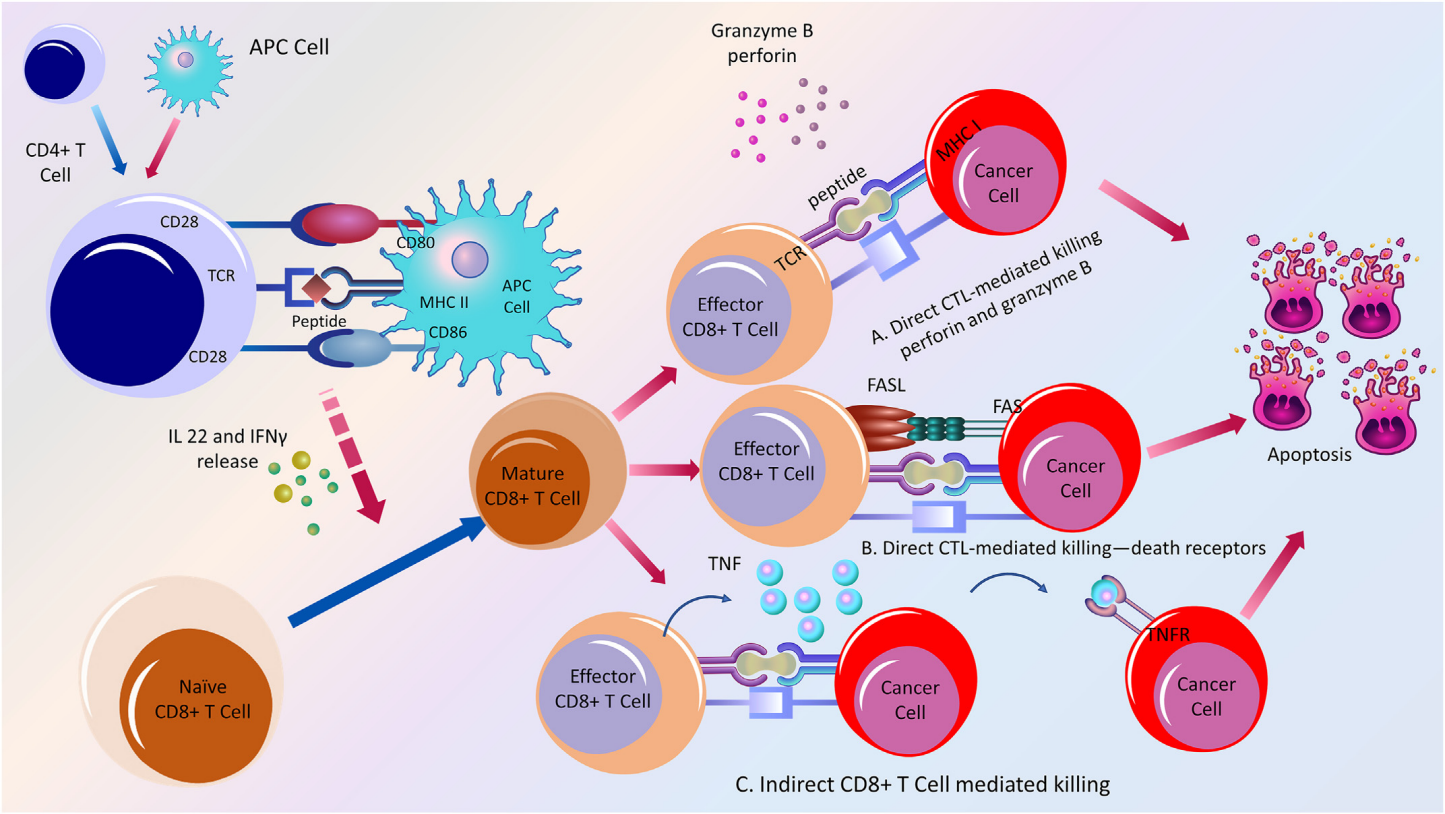
Action of CD8+ T cells in cancer.CD8+ T cells, or CTLs, are frontline immune cells employed in cancer and cancer immunotherapy. They kill potentially harmful cells in the body, including tumor/tumor-like cells, viruses, and foreign antigens. The activation and proliferation of CD4+ T cells and their interaction with APCs result in IL-22 release. IFN-γ helps naïve CD8+ T cells to convert to mature CD8+ T cells. Effector CD8+ T cells are produced from mature CD8+ T cells and are short-lived. They can kill tumors or tumor-like cells either directly or indirectly. (A) Direct CTL-mediated killing via perforin and granzyme. This necessitates a cell-to-cell interaction, accompanied by the release of cytolytic enzymes, such as granzyme B. Perforin released by CTL creates pores in a juxtaposed cancer cell membrane, enabling passive inward diffusion of granzyme B. This causes the targeted cells to undergo apoptosis. (B) Direct tumor cell killing can occur due to an association between the Fas-L, expressed by CTL, and its receptor, Fas, represented by cancer cells. Fas/Fas-L ligation induces cancer cell apoptosis through a caspase-dependent pathway. (C) Indirect CD8+ T cell-mediated killing: CTLs can cause indirect or “bystander” tumor cell death by secreting cytokines that act at a distance. TNF-α secretion can trigger apoptosis in tumor cells that express the TNF receptor. APCs: Antigen-presenting cells; CTLs: Cytotoxic T lymphocytes; Fas-L: Fas ligand; IFN-γ: Interferon-γ; IL: Interleukin; TNF: Tumor necrosis factor.

CD8 is a transmembrane glycoprotein that is expressed on CTLs. They play a vital role in anticancer immunity and are the cornerstone of effective cancer immunotherapy.^73^ CAR, a type of genetically reconstructed receptor, is used with CD8+ T cells during ACT. These procedures have significantly impacted immunotherapy against numerous types of cancer.^73^^,^^74^ Currently, immune checkpoints and pathways, in addition to CAR T cells, are being assessed in clinical experiments.^75^ CD8+ cytotoxic T lymphocytes are superficially attached to antigenic peptides that introduce APCs or other desired cells and link MHC-1 molecules to destroy target antigens or cells [Figure 2].^73^^,^^76^ Furthermore, CD8+ T cells interact with tumor cells and initiate apoptosis via pore generation with the help of mechanical force: they merge death-inducing granules containing granzymes, cathepsin C, and perforin.^77^

Pores can also be formed in target or tumor cells via perforin and granulysin endocytosis. Perforin and granulysin form a porous endosomal membrane, which enables the delivery of various granzymes to the cytoplasm.^78^ Moreover, CD8+ T cells express the Fas ligand, which binds to the Fas receptor on target cells and triggers caspases and endonucleases. This results in target cell DNA segmentation and activates Fas-associated death domains [Figure 2].^79^ Monoclonal antibodies are linked to immune-oncology via checkpoint inhibition and amelioration of the clinical consequences of various cancers.^80^ This approach boosts the potency of the immune response against tumors and revitalizes debilitated CD8+ T cells. To segregate and augment tumor-determined CD8+ T cells, a large proportion of T cells is collected from peripheral blood to construct CAR T cells. This strategy has the potential to revolutionize oncology.^81^

## T cell metabolism in cancer immunotherapy

The TME shows metabolic hindrance against anti-tumor T cell functionality. This metabolism synchronizes the functionality and pre-destination of T cells. Therefore, utilizing metabolic knowledge may help ameliorate T cell-based immunotherapy.^82^ T cells attack pathogens and constantly scrutinize and destroy tumor cells.^8^ CPI molecules and the TME are metabolically ambivalent and halt anti-tumor function. Antibody-mediated immune checkpoint therapy ameliorates T cell responses in cancer; however, these therapies failed in large-scale studies. Moderately exhausted T cells have shown faulty metabolic outcomes; therefore, immune therapies such as PD-1 blockade have become complex.^83^^,^^84^ Glucose assimilation is reduced because PD-1 and CTLA-4 receptors hinder the glycolytic pathway and impede T cell activation.^85^ In contrast, PD-1 induces fatty acid oxidation (FAO) and lipolysis.^82^^,^^85^ A study conducted in mice showed that blocking PD-1 can drive back glucose restriction in TILs and ameliorate glycolysis through mTOR signaling, and induction of IFN-γ reproduction enhances CD8+ T cell effectiveness against tumors.^86^

Inhibition of glycolysis through 2-deoxyglucose ameliorates the functionality of CD8+ T cells against tumor cells and memory cells because long-term uncontrolled glycolysis may result in T cell exhaustion.^87^ Additionally, protein kinase B (PKB) inhibitors promote lipid oxidation metabolism, fetch up CD4+ T cell markers, such as SRC, and ameliorate tumor-specific lymphocytes.^88^ The activity of mitochondria is reduced in tumor cells because of peroxisome proliferator-activated receptor gamma coactivator one alpha (PGC1α) hindrance, arbitrated by PKB.^89^ Enhancing the functionality of mitochondria and rendering them resistant to oxidative stress by increased PGC1α expression makes PGC1α a promising therapeutic target.^89^^,^^90^ The efficacy and functionality of PGC1α-activating agents are yet to be determined; however, they may facilitate T cell functions against tumors.^82^

## Memory T cell in cancer immunotherapy

Antigen-mediated T cells provide adequate immune protection against various human cancers.^91^ The memory of T cells was first recognized when it was revealed that T cells could be subdivided into many kinds depending on the synthesized molecules and chemokines receptors expressed on their surface.^92^ The phenotype distinction of central memory T cells (Tcm) converts into distinctive migratory characteristics. These cells instantly circulate much like naïve T cells through blood flow to lymphoid organs, where Tem is constantly transferred to the non-lymphoid tissue.^93^ Recent findings have demonstrated that resident memory T cells (T_rm_s) could be categorized based on their respective organs.^94^ This novel class of CD 8+ T memory cells in both animal models and humans can be identified by their phenotype (CD103+, CD69+); they do not recycle in the blood and are involved in the protective immune reactions against pathogens.^95^ As an important player in cancer immunology, their role in cancer has recently emerged.

**Figure 3:**
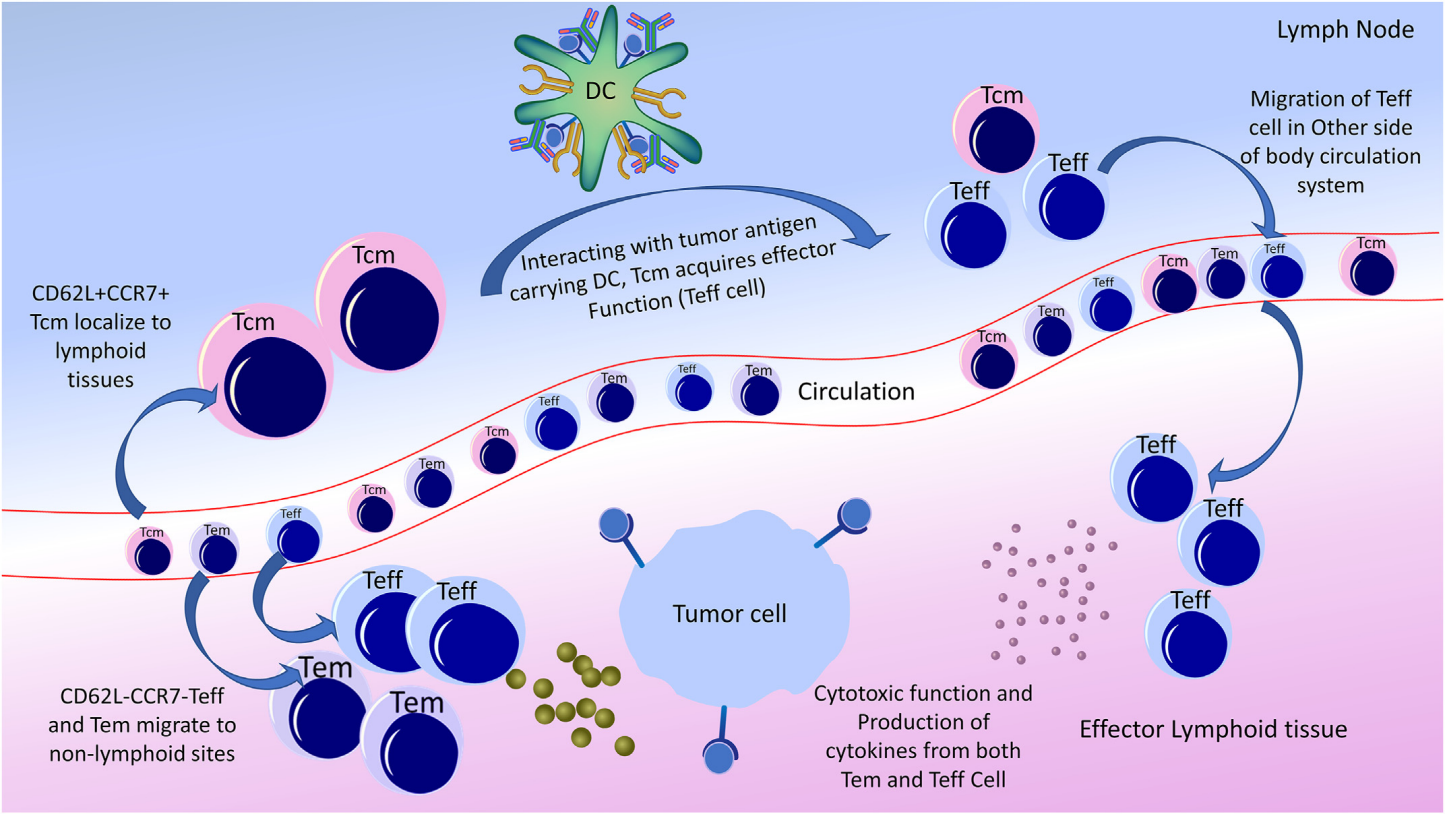
Schematic presentation of CD8+ T cell population in cancer immunotherapy. Mature CD8+ T cells are subdivided into three groups based on their use and activity: Tcm, Tem, and Teff. These cells have essential and complementary roles in the immune system against tumors and foreign antigens. For example, CD62L is expressed by Tcm, which predominantly migrates to nearby lymphoid tissue and reacts with tumor antigen-presenting DCs. DCs: Dendritic cells; Tcm: Central memory T cells; Teff: Effector T cells; Tem: Effector memory T cells.

T_rm_s are present in many human cancers and are associated with favorable clinical outcomes independent of CD8+ T cell invasion.^96^ In 1999, two separate populations of polyclonal CD81 CD45RA2 memory T cells were identified in the peripheral blood of humans, which were designated based on their expression of the lymphoid homing markers C–C chemokine receptor 7 (CCR7) and CD62L as Tcm and Tem, respectively.^92^ The presence of these two distinct subsets of memory T cells implies that each must play a significant role in maintaining protective immunity.^97^^,^^98^ The significance of CD62L2 in combating localized infections is well established.^99^^,^^100^ This coincides with the fact that Tem is more active than the phenotype. They express adhesion molecules and chemokine receptors involved in homing lymphoid effector tissue and inflammatory sites and are ready to react quickly to peripheral problems.^101^^,^^102^ Tcm is vital for defense against systemic high-pathogen-load infections.^100^^,^^102^ Because CD62L1 Tcm is preferred in lymphoid tissues rather than Tem and has improved survival benefits and the ability to increase, they also have antigen-specific T cells, which can spread and fill the periphery with a secondary challenge.^103^ These subsequent events allow Tcm to extend, acquire effector functions, and return to the tumor site. In contrast, Tem and Teff migrate to the peripheral tissues and develop effector lymphoid tissue, resulting in immediate cytotoxic activity at the tumor site and consequent rapid tumor death. While the various functions of Tcm and Tem in infection immunity are well accepted, there is still much debate about the most beneficial type of memory T cells for the growth and maintenance of anti-tumor immunity. As Tcm expands well and persists for a long duration in response to secondary activation, it is expected to protest against large tumors and metastasis. In comparison, Tem can perform immediate cytotoxic actions and act quickly to stop the growth of emerging tumors at isolated peripheral residues [Figure 3]. Whether one of these memory cell subsets is superior in providing cancer-protective immunity is not clearly understood. There is a plethora of evidence supporting both Tcm and Tem as major players in tumor-protective immunity.

## T cell dysfunction in cancer immunity and immunotherapy

T cells play a significant role in diverse immune responses in cancer, autoimmune diseases, and multiple chronic infections. T cells are rapidly activated into effector T cells during various infections in humans. Effector T cells (Teffs) participate in the clearance of antigens, and most of the Teffs die after cleaning the antigens. A small number of Teffs are converted into memory T cells, which respond quickly when the same or similar antigen reappears.^104^ During various chronic infections and cancers, T cell function becomes complicated, and T cell dysfunction usually occurs due to exposure to persistent antigens.^103^ Recent studies have found that T cell dysfunction is related to the level of antigen stimulation.^105^^,^^106^

TCR-dependent pathways are associated with T cell dysfunction.^107^ Furthermore, chronic antigen stimulation is responsible for persistent exposure to PD-1, which controls the level of TCR signaling.^108^^,^^109^ Therefore, persistent characteristics and the level of antigen stimulation are considered significant factors that lead to T cell dysfunction. These factors are also related to the severity of T cell dysfunction. Another reason for T cell dysfunction is the exhaustion of T cells. Exhausted T cells (Tex) usually function differently than dysfunctional T cells.

T cell dysfunction was first identified in lymphocytic choriomeningitis virus (LCMV) and is observed by the continuous loss of functions, such as proliferation, cytokine production, and efficiency to lyse the target cells.^110^ Moreover, T cell dysfunction has been observed in humans due to cancer and various chronic diseases.^111^^,^^112^ Dysfunctional CD8+ T cells in humans and mice were found to co-regulate various inhibitory receptors (IRs). The severity of T cell dysfunction^113^ is related to the level and number of IRs. Notably, dysfunctional T cells were less helpful but fell to eliminate infection and cancer with good efficacy.

## T cell exhaustion

T cells have persistent antigens during chronic infections and cancers. This function is related to the deterioration of T cells, termed ‘exhaustion of T cells.’ The exhausted T cells were found to lose their effector functions and alter their transcriptional program. Other features include the progressive loss of effector function and co-expression of multiple IRs.^114^^,^^115^ T cell exhaustion is associated with poor or less control to eliminate persistent infections and tumors. In acute infections and vaccinations, naïve T cells are activated and converted into effector T cells within 1–2 weeks.^116^^,^^117^ After clearing the antigens, most of the activated T cells (about 90%–95%) die through the apoptosis pathway. However, few persisting T cells are converted into memory T cells. Memory T cells downregulate most of the effector T cell activation events. They can also be effectively reactivated by effector functions.^117^

Moreover, memory T cells possess a significant property of antigen-independent self-renewal, which is a stem cell-like and slow division property guided by IL-7 and IL-15. An essential point in the development of memory T cells is that, after the effector, state memory development occurs if there is no continuous antigen stimulation and a higher rate of persistent inflammation. When the same or similar antigens are exposed, memory T cells expand rapidly and gain more effector functions than naïve T cells.^114^^,^^115^ This feature permits T cells to persist and provide protective immunity for a long time, even when antigens are removed. In contrast, in various chronic infections and cancers associated with persistent antigen exposure, the differentiation of memory T cells is altered.

**Figure 4:**
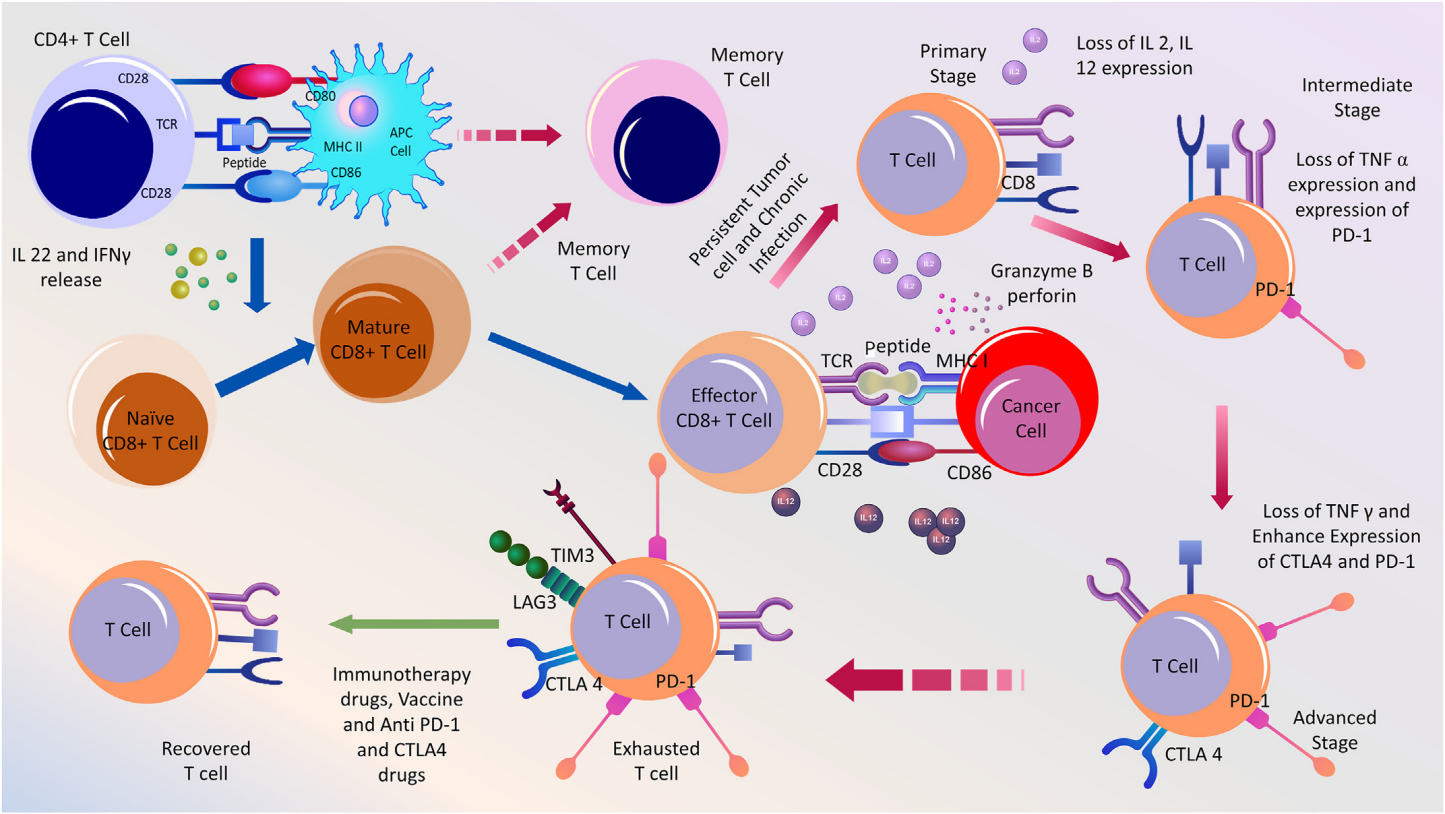
A brief presentation of T cell exhaustion events in cancer.Both CD8+ and CD4+ T cells produce memory T cells to prevent recurrent invasion by the same antigenic component. In chronic and persistent infections, immune cells become hyperactive and exhausted. This can lead to a reduction in the T cell secretion of IL-2 and IL-12 in the primary stage. This condition can worsen when T cells begin to express PD-1. In addition, TNF-α secretion was reduced. The situation worsens when TNF-γ secretion is reduced and another immune checkpoint, CTLA-4, is expressed on the T cell surface. The T cells were exhausted and ready to die. It represents two more receptors on its surface, TIM-3 and LAG-3. This exhausted condition can be recovered through anti-PD-1 and CTLA-4 pathway drugs, vaccines, and immunotherapy. CTLA-4: cytotoxic T-lymphocyte-associated protein 4; IL: Interleukin; LAG-3: Lymphocyte-activation gene-3; PD-1: Programmed death receptor-1; TIM-3: T cell immunoglobulin domain and mucin domain 3; TNF: Tumor necrosis factor.

T cell exhaustion was first observed in mice during chronic infections^111^^,^^118^; it was found in humans during chronic infections with human immunodeficiency virus (HIV), hepatitis C virus (HCV), and cancer.^114^^,^^119^ IL-2 production is lost during the primary state of exhaustion. Moreover, the production of TNF-α and IFN-γ is lost in the intermediate and advanced states of exhaustion, respectively.^103^ T cell exhaustion inhibits the proper monitoring of infection. PD-1 is considered the key inhibitor receptor for T cell exhaustion because T cells with high PD-1 expression lose the ability to effectively eliminate cancer. Therefore, reversal of T cell exhaustion is considered a promising strategy for treating cancer [Figure 4]. The PD-1/PD-L1 pathway, a critical factor in T cell exhaustion, has been found to work effectively in cancer treatment. However, further studies are needed to understand the mechanism of PD-1/PD-L1 action in overcoming T cell exhaustion in cancer and chronic infections.

Nonetheless, exhausted T cells manifest high levels of CTLA-4, lymphocyte activation gene 3 protein (LAG-3), T cell immunoglobulin domain, and TIM-3.^120^ The immune checkpoint receptor CTLA-4 manifests only on T cells that attach to CD80/CD86 ligands and induce intracellular inhibitory signal transduction.^121^ CTLA-4 interacts with CD80/CD86, inhibiting T cell stimulation and IL-2 and IL-12 synthesis^122^ PD-1+ CTLA-4+ CD8+ TILs have been more adversely exhausted in proliferation and cytokine synthesis as PD-1 and CTLA-4 are co-expressed in one-third or half of the CD8+ TILs, but combined inhibition of PD-1 and CTLA-4 improves T cell activity in cancer [Figure 4].^123^

As PD-1 and TIM-3 are co-expressed in tumor-containing animal models, TIM-3+ PD-1+ CD8+ TILs are perhaps the most prevalent subgroup and demonstrate more exhausted characteristics than both TIM-3- PD-1- and TIM-3+ PD-1+ CD8+ TILs, which fail to synthesize TNF-α, IFN-γ, and IL-2.^124^ In this case, the anti-tumor activity of exhausted CD8+ T cells was restored by blocking both PD-1 and TIM-3.^124^ In another animal study, tumor cells co-expressing PD-1/LAG-3 demonstrated more exhausted characteristics than positive TILsr-negative TILs alone.^125^ Blocking both PD-1 and LAG-3 may result in tumor retrogradation.^125^

Taken together, these data show that PD-1 is a key molecule involved in T cell exhaustion. In addition, the sequence of IR co-expression in CD8+ T cells influences the intensity of T cell exhaustion. Therefore, combined receptor-blocking therapy may be a unique solution for cancer treatment.

## Conclusion and future directions

The studies summarized in this review have shown the crucial function of T lymphocytes in cancer immunotherapy. Their use in cancer treatment has great potential because these cells can specifically target tumors. Promising strategies induce cytotoxic T cells to act against cancer cells. However, like other conventional therapies, T cell-based therapies may cause several side effects. For example, therapies against CTLA-4 may have shown superior efficacy in some individuals; however, they may lead to severe autoimmunity^126^ and be less efficacious than PD-1-PD-L1 inhibitors.

Nevertheless, CTLA-4 and PD-1-PD-L1-targeted combined therapy might play an influential role in the treatment of tumors. The correct doses at the right time should be applied to impede tumor maturation, which requires further optimization. In addition, immunotherapies targeting CTLA-4 may be used as complementary strategies to chemotherapy and radiation therapy; such combined strategies might yield promising results.

Determining the type of tumor is crucial to increase the potency of integrated therapy. Additionally, it is important to determine the most suitable immunotherapy mode. Currently, the use of CAR T cell therapy is restricted to young individuals and children with lymphoblastic leukemia who respond poorly to chemotherapeutic strategies. Contemporary solid tumor manifestations have been revolutionized by clustered regularly interspaced short palindromic repeats and -associated protein 9 (CRISPR/Cas9) gene-editing technology, which has derived the latest cohort of CARs.^76^ The major bottleneck of CAR T cell therapy is that these cells cannot distinguish between tumor cells and normal cell-surface proteins, which might lead to cross-reactivity and severe illnesses. Integrated administration of immune checkpoint inhibitors and other anticancer drugs may improve survival in patients with cancer where immune checkpoint inhibitors are ineffective.^127^

## Funding

This research did not receive any specific grant from funding agencies in the public, commercial, or not-for-profit sectors.

## Author contributions

All authors confirm that the concept, design, data collection, analysis and interpretation, and writing are our own. We ensured that no other contributors were present.

## Ethical approval

None.

## Data availability statement

All data are available within this manuscript.

## Conflict of interest

None.
